# Endomyocardial fibrosis and apical calcification: A case report with unusual presentations of apical hypertrophic cardiomyopathy

**DOI:** 10.1097/MD.0000000000035823

**Published:** 2023-11-10

**Authors:** Man Gao, Feifei Zhang, Yuetao Xie, Jialin Li, Xiao Hao, Huiliang Liu, Xiaoyong Qi, Yi Dang

**Affiliations:** a Department of Cardiology, Hebei General Hospital, Shijiazhuang, China.

**Keywords:** apical calcification, apical hypertrophic cardiomyopathy, endomyocardial fibrosis, hypertrophic cardiomyopathy, ventricular thrombus

## Abstract

**Rationale:**

Apical hypertrophic cardiomyopathy (ApHCM) is a phenotypic variant of hypertrophic cardiomyopathy. Endomyocardial fibrosis and endocardial calcification are especially rare in ApHCM.

**Patient concerns:**

The main symptoms was chest tightness, palpitation, shortness of breath, and fatigue. Echocardiography and imaging examinations found apical hypertrophy along with endocardial calcification and endomyocardial fibrosis. Abnormal structural changes led to thrombosis and made the left ventricle a flat shape resembling an “apple.”

**Diagnoses:**

The typical presentations, hypertrophic apex on echocardiography and an elevated N-terminal pro-brain natriuretic peptide level indicated the diagnosis of ApHCM and heart failure with preserved ejection fraction.

**Interventions:**

Optimal medical therapy including the administration of ApHCM, heart failure and atrial fibrillation to improve symptoms and life quality.

**Outcomes:**

Since discharge, the patient could perform normal daily activities and had no discomfort based on the optimal medical therapy.

**Lessons:**

We report a ApHCM patients with unusual presentations of endomyocardial fibrosis and apical calcification. This case highlights the importance of understanding the specific pathological changes of ApHCM for treatment and prognosis.

## 1. Introduction

Apical hypertrophic cardiomyopathy (ApHCM) was first described in Japan in 1976, which is a phenotypic variant of hypertrophic cardiomyopathy (HCM) characterized by apical hypertrophy, restrictive ventricular filling and giant T wave inversion.^[1,2]^ It is not as rare as previously reported with an incidence of 25% in Asian populations and 3% to 10% in non-Asian populations.^[3]^ Differing from classical HCM, the main pathophysiological features of ApHCM are midventricular obstruction with cavity obliteration and apical aneurysm formation without left ventricular (LV) outflow tract obstruction and mitral regurgitation.^[2]^ ApHCM is inherited in an autosomal dominant mode, whereas only a quarter can be tested positive for a carrier gene.^[4]^ Endomyocardial fibrosis and apical calcification can result from lots of etiologies, which is uncommon in ApHCM. Here, we report a rare case of ApHCM accompanied by endomyocardial fibrosis (EMF) and apical calcification, and further discuss the involved potential mechanisms.

## 2. Case presentation

A 57-year-old female patient presenting with chest tightness, palpitation, shortness of breath, and fatigue was admitted to our hospital in March 2022. In the past 3 years, the above symptoms intermittently occurred and gradually worsened, meanwhile the activity endurance decreased obviously. There was a history of myocarditis, paroxysmal atrial fibrillation, and 2-type diabetes for more than 10 years. No travel history or contact history with the epidemic area. No smoking or alcohol addiction. No family history. At admission, the patient had a blood pressure of 144/73 mm Hg, a pulse of 70 beats/min, and a body mass index of 24 kg/m^2^. Physical examination showed a regular heart rhythm, a lower heart sound, and no pathological murmur in the area of cardiac valves. No jugular venous distention and moist rales in the lungs were found, but mild edema of the lower extremities was present.

Laboratory results showed a higher amino-terminal pro-B-type natriuretic peptide (N-terminal pro-brain natriuretic peptide 2169 pg/mL) and D-dimer quantification(3.17mg/L). Liver function revealed that the total bilirubin was 53.3 μmol/L, the direct bilirubin was 8.9 μmol/L and γ-glutamyltransferase was 172.1 U/L. Renal function showed that urea was 8.2 mmol/L, creatinine was 104.7 μmol/L, and the estimated glomerular filtration rate was 51.10 mL/min. The routine blood tests did not reveal elevated inflammatory markers or an increased eosinophil count. Virus antibody detection showed that there were no recent viral infections. Immune-related indicators such as erythrocyte sedimentation rate, C-reactive protein, rheumatoid factor, vasculitis antibody, and antinuclear antibody were not abnormal. Electrocardiogram on admission presented sinus rhythm, poor R-wave progression in the chest leads, and abnormal ST-T change. During hospitalization, 24h-Holter found that there was paroxysmal atrial fibrillation and second-degree sinoatrial block.

**Figure 1.**
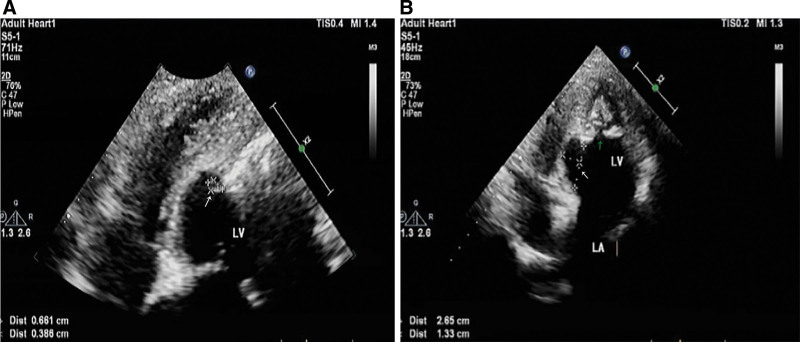
Echocardiography showing ventricular thrombus (A, white arrow), ventricular septal aneurysm (B, white arrow), apical hypertrophy and calcification (B, green arrow).

**Figure 2.**
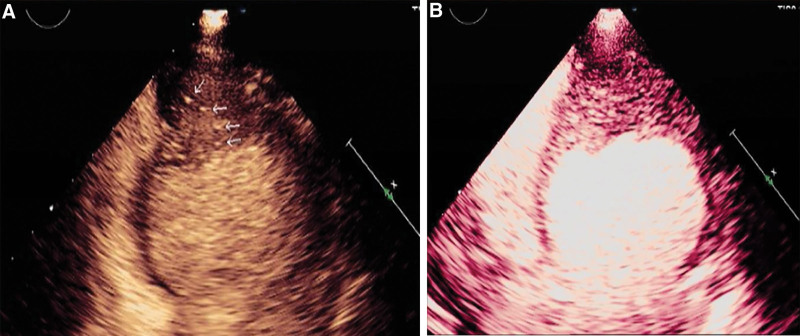
Contrast echocardiography showing apical calcification (A, white arrow) and the “apple” sign of the morphology of the left ventricular chamber with contrast agent filling (B).

**Figure 3.**
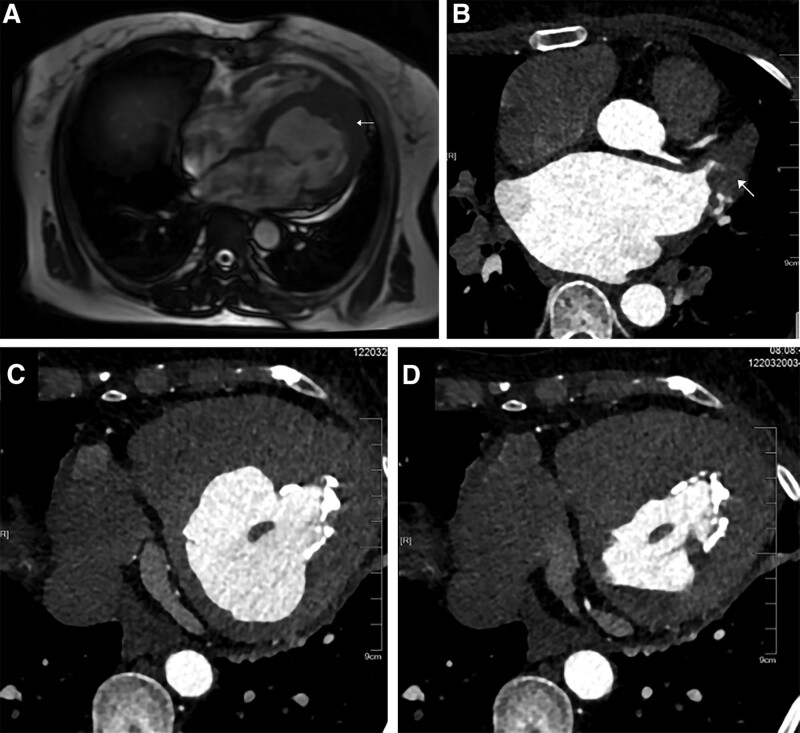
Cardiac CT showing apical calcification (A, white arrow), contrast-enhanced imaging showing apical hypertrophy and filling defect (B, white arrow), and limited left ventricular filling during diastole and systole (C, D). CT = computed tomography.

**Figure 4.**
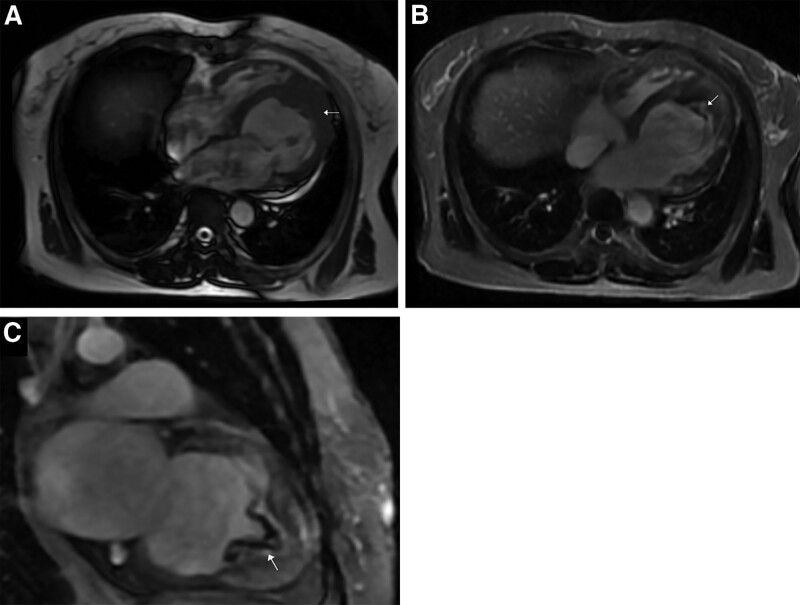
Cardiac magnetic resonance imaging showing apical hypertrophy (A, white arrow) and late gadolinium imaging indicating endomyocardial fibrosis of the left ventricle (B and C, typical “double V” sign, subendocardial enhancement with overlying thrombus).

A transthoracic echocardiography indicated left heart dilation, LV apical hypertrophy (approximately 20 mm), and endocardial calcification at the apex. There was ventricular thrombus ranging about 6.6 × 3.8mm on the endocardial surface in the middle and lower parts of the posterior ventricular septum (Fig. 1A). The middle and upper segments of the ventricular septum were thin and showed a tumor-like bulge direction to the right ventricle during diastole with a range of about 26.5 × 23.3 mm (see Fig. 1B). The LV ejection fraction measured by Simpson method was 54%. Contrast echocardiography of left heart showed that the LV cavity was oblate, and the gap-like contrast agent was filling at the apex which looked like an “apple”(see Fig. 2). There was little movement at the apex, conversely, the motion of the middle and lower ventricular septum was enhanced. Computed tomography (CT) of the coronary artery found no obvious stenosis, but apical calcification and myocardial hypertrophy (see Fig. 3). Cardiac magnetic resonance (CMR) imaging and delayed enhancement findings were indicative of wall thickening in the apical segment (approximately 15 mm) with flaky high-signal shadow. Arc-shaped delay of high-signal shadow in the apical and middle segment of the ventricular septal was considered endocardial fibrosis. There were small strips of low-signal shadow in the endocardium of the apex suggesting calcification (see Fig. 4). Genome sequencing results revealed that there were missense mutations in the coding region of the MYH7 gene and GATA6 gene.

During hospitalization, the patient occurred transient limb weakness and motor aphasia. Physical examinations found gaze of eyes to the right, grade IV of the limb muscle strength, and normal muscle tension. However, these presentations gradually recovered for several minutes. Subsequently, there were no bleeding and hematoma detected by the cranial CT, as well as no new cerebral infarction tested by magnetic resonance. Magnetic resonance angiography showed multiple localized stenosis or occlusion of cerebral vessels. Most possibly, the patient occurred a transient ischemic attack caused by microthrombosis from left heart.

According to the clinical manifestations, laboratory results, and imaging examination findings, ApHCM and heart failure with preserved ejection fraction was diagnosed. Meanwhile, endocardial fibrosis, LV mural thrombus and apical calcification were present. The patient was treated with angiotensin receptor neprilysin inhibitor, β-receptor blocker, loop diuretics, and aldosterone receptor antagonist based on the guidelines of heart failure. Sodium-dependent glucose transporters-2 inhibitor was used for the administration of 2-type diabetes, also had cardiac benefits. Reducing the ventricular rate and preventing thrombosis had been the predominant regimens for intermittent episodes of atrial fibrillation. Considering that the patient had an LV mural thrombus and suffered a transient ischemic attack during hospitalization, rivaroxaban, a new oral anticoagulant, was recommended for the treatment of paroxysmal atrial fibrillation and ventricular thrombus. As for ApHCM, apical myectomy can be another option if the symptoms were difficult to control with medication, or heart transplantation can be finally considered.

Through telephone follow-up, we learned that the patient’s symptoms and life had improved significantly with simple daily physical activities since discharge. A long-term follow-up would be continued on this patient.

## 3. Discussion

ApHCM is a phenotypic variant of hypertrophic cardiomyopathy in which hypertrophy is localized to the apex with or without midsegment involvement. It is characterized by diastolic dysfunction with increased ventricular filling pressures and left atrial enlargement.^[2]^ This case had typical features of diastolic heart failure with apical hypertrophy and limited diastolic filling. The findings of auxiliary examinations showed that a series of changes developed in the cardiac structure and movement, including LV thrombus, apical calcification, EMF, ventricular septal aneurysm, abnormal wall motion, and restricted diastolic function. The pathophysiological mechanisms underlying these changes warrant further discussion.

Previous reports^[5,6]^ had described apical calcification in ApHCM. Myocardial calcification can be caused by several etiologies. There are 2 main types: dystrophic calcification and metastatic calcification. Dystrophic calcification occurs in local damaged tissue and cellular necrosis, such as myocardial infarction, ventricular aneurysm, myocarditis, EMF, immune diseases, tuberculosis, and rare cardiac tumors. Metastatic calcification is associated with abnormal calcium metabolism occurring in normal or diseased tissues, including renal failure, hyperparathyroidism, and vitamin D-related disorders.^[7]^ This patient presented with EMF and suffered myocarditis previously. Additionally, abnormal blood flow gradients in patients with ApHCM are prone to apical thrombosis, which may also calcify over time.^[8]^ Hence, apical calcification, in this case, was speculated as dystrophic calcification possibly associated with EMF, myocarditis, old thrombus, or confounding factors.

Endomyocardial fibrosis (EMF) is characterized by restricted diastolic filling in the ventricles resulting from the deposition of fibrous tissues on the surface of the endocardium.^[9]^ EMF is a rare disease that occurs mainly in tropical countries leading to severe heart failure.^[10,11]^ It may be connected with poverty, malnutrition, parasitic infestation, genetics, and ethnicity. Eosinophilia and infection are thought to be potential major triggers.^[10]^ Secondary EMF usually exists in patients with underlying ischemic heart disease and primary myocardial disease.^[12]^ CMR can accurately identify EMF with the typical “double V” sign consisting of normal myocardium, fibrotic endocardial myocardium and covered thrombus.^[13]^ This patient did not involve any above conditions related to tropical EMF including infection and eosinophilia. It might be secondary to the ischemia background in ApHCM.

It is shown that myocardial ischemia can occur in ApHCM without any epicardial atherosclerotic disease, which involved abnormal coronary small vessels, reduced arteriolar density, as well as myocardial bridging(squeezing action by hypertrophic myocardium during systole).^[14]^ Furthermore, the increased ventricular filling pressure can result in a decrease in microvascular perfusion pressure. These hemodynamic effects may lead to a pathophysiological cascade of infarction, fibrosis, and dystrophic calcification.^[5]^ EMF and myocardial calcification in this case further limited the wall motion and ventricular diastolic filling, ultimately developing into severe heart failure. Changes in the structure of the cardiac chamber, such as hypertrophic calcified apex and ventricular septal aneurysm, would result in stasis of blood flow, which predispose to thrombus formation.

Genetic testing may not be informative to diagnosis and treatment, but it is of great significance for early screening and risk assessment in families. In a large genetic testing cohort for HCM, ApHCM was present in <10%, and only 25% had a positive genetic test result. The most common gene mutation types were MYBPC3 and MYH7.^[4]^ We conducted genetic testing for the patient in this case and her daughter. The results showed that there were missense mutations in the coding region of the MYH7 gene and GATA6 gene.

Medical therapy in ApHCM is similar to typical HCM. It is worth noting that an anticoagulant is necessary for patients accompanied by atrial fibrillation or apical aneurysms in ApHCM. Septal ablation is not recommended in patients with ApHCM unless there is obvious septal hypertrophy leading to LV outflow tract obstruction, while catheter ablation is reserved for apical scar-related ventricular tachycardia. Apical myectomy has already been confirmed to increase end-diastolic diameter and improve symptoms. Besides, ventricular assist devices and heart transplantation remain therapeutic options for refractory heart failure or intractable life-threatening arrhythmias.^[2,15,16]^

## 4. Conclusion

Apical hypertrophic cardiomyopathy is uncommon in all heart diseases. Endomyocardial fibrosis and apical calcification increase the difficulty of diagnosis and treatment of ApHCM, which can be identified early by imaging examination, especially CMR. An accurate and comprehensive understanding of ApHCM and its underlying pathophysiological mechanisms is significant for treatment and prognosis.

## Acknowledgments

We thank the patient and her family for giving us permission to share this rare case, and express our gratitude to the imaging department and ultrasound department for providing us with imaging data.

## Author contributions

**Formal analysis:** Feifei Zhang.

**Investigation:** Yuetao Xie, Xiaoyong Qi, Yi Dang.

**Methodology:** Jialin Li.

**Project administration:** Huiliang Liu.

**Resources:** Xiao Hao.

**Validation:** Yi Dang.

**Writing – original draft:** Man Gao.
